# Dutch Society of Infection Prevention in Healthcare (VHIG)

**DOI:** 10.1186/1753-6561-5-S6-P2

**Published:** 2011-06-29

**Authors:** T de Ruiter, GG Van Knippenberg-Gordebeke

**Affiliations:** 1 Infection Prevention & Hygiene, Groene Hart Hospital,, Gouda, Netherlands; 2 KNIP Consultancy Infection Prevention, Venlo, Netherlands

## Introduction / objectives

The Dutch Society of Infection Prevention in Healthcare society was founded in 1973 and is since 1987 member of the International Federation of Infection Control (IFIC). VHIG has currently 350 members entitled: Consultant Infection Prevention (CIP).

## Methods

Supporting the members in carrying out their task in all healthcare settings and disseminating information about the prevention and control of infections. The VHIG attempts to achieve its objectives by means of: giving information, instructions, advice, conferences, lectures and publications. VHIG takes care of Accreditation and re-registration every 5 year for all the members if they fulfil the requirements.

## Results

Since 2006 the Dutch advice is 1 FTE CIP per 5000 admissions, or 1 / 178 hospital beds, which cannot fulfilled for different reasons. Dutch hospitals are committed to pursuing active infection control policies based on the Working group Infection Prevention (WIP). These guidelines are considered as professional standards by the Healthcare Inspectorate. The CIP gives shape to this standard. The professional background of the CIP is Nurse, Lab Technician, and Epidemiologist who graduate with a diploma after an in-service Post-Bachelor training of 18 month.

## Conclusion

With the Dutch Society for Microbiology the VHIG drafted in 2008 a guideline for quality assurance to achieve continuous quality improvement *‘Kwaliteitsrichtlijn voor Infectiepreventie in Ziekenhuizen’* The document is based the ISO 9001:2000 series and the derived Directive for Healthcare.

NPR-CEN/TS 15224:2005. The VHIG try to implement the Recommendations of the Council of European Union on patient safety, including the prevention and control of healthcare associated infections (*File:2009/0003* (*CNS.* Hand hygiene and Surveillance surgical side infections are continuous on the agenda.

## Disclosure of interest

None declared.

